# Vegetable By-Products as Alternative and Sustainable Raw Materials for Ruminant Feeding: Nutritive Evaluation and Their Inclusion in a Novel Ration for Calf Fattening

**DOI:** 10.3390/ani13081391

**Published:** 2023-04-18

**Authors:** Irantzu Goenaga, Aser García-Rodríguez, Idoia Goiri, Sara León-Ecay, Joana De Las Heras, Noelia Aldai, Kizkitza Insausti

**Affiliations:** 1 Tratamiento Subproductos Agroalimentarios, S.L. (TRASA), Camino San Juan s/n, 31320 Milagro, Spain; 2 Institute of Innovation and Sustainable Development in Food Chain (IS-FOOD), Higher Technical School of Agricultural Engineering and Bioscience, Campus de Arrosadía, Universidad Pública de Navarra (UPNA), 31006 Pamplona, Spain; 3 NEIKER—Basque Institute for Agricultural Research and Development, Basque Research and Technology Alliance (BRTA), Department of Animal Production, Campus Agroalimentario de Arkaute s/n, 01192 Arkaute, Spain; 4 Lactiker Research Group, Department of Pharmacy and Food Sciences, University of the Basque Country (UPV/EHU), Paseo de la Universidad 7, 01006 Vitoria-Gasteiz, Spain

**Keywords:** circular economy, vegetable by-products, agri-food industry, ruminant feed, nutritive value, digestibility, gas production, rumen fermentation

## Abstract

**Simple Summary:**

Large amounts of vegetable by-products are generated by the agri-food industry every day. Giving them a second life can be positive for the producing companies, with a special emphasis on improving their sustainability indicators. In addition, vegetable by-products are promising raw materials for ruminant nutrition. In this study, first, we evaluated the nutritional composition, in vitro digestibility, and gas production kinetics of 15 vegetable by-products using corn silage as the positive control. From these results, the second part of the research was focused on the formulation of two different diets for fattening calves: a conventional ration based on concentrate and straw and an alternative one based on vegetable by-products. Most vegetable by-products revealed greater nutritive value than corn silage and results indicated that their inclusion in finishing cattle diets may reduce the use of other ingredients without impairing ruminal fermentation.

**Abstract:**

This research aimed to evaluate the nutritional composition, in vitro digestibility, and gas production kinetics of 15 vegetable by-products generated by the agri-food industry compared with corn silage as a reference raw material. Nutritional characterization and in vitro ruminal fermentation tests were performed to determine in vitro organic matter digestibility and digestible energy values, short-chain fatty acids, and the gas production profile. Results indicate that vegetable by-products were more degradable, more extensively fermented, and fermented at a faster rate than corn silage. Going one step further in the valorization of these by-products in animal feed, the second part of the research aimed to compare the novel ration designed for calf fattening with a conventional one. An artificial rumen unit was used to obtain nutrient disappearance, rumen fermentation parameters, and gas production of rumen digesta. Very slight differences were observed between both experimental rations, with their composition being the main difference. Most of the unitary vegetable by-products and all mixes, as real examples of by-product generation in the agri-food industry, have higher digestibility and a greater nutritional value than corn silage. These by-products showed the potential to be used in ruminant-ensiled rations and could replace part of the ingredients in conventional diets.

## 1. Introduction

Today, promoting the sustainability of the production chain is no longer a choice. This fact is reflected in the new strategies adopted by the European Union (EU), which are centered on reducing food loss and waste, with the main objective of obtaining a sustainable food chain in addition to producing new sustainable food and feed sources. A clear example can be found in the Sustainable Development Goals, as it aims at the target 12.3 to halve food wastage per capita worldwide by 2030 [1].

The food and drink industry is a major contributor to Europe’s economy, generating a turnover of EUR 1093 billion and an added value of EUR 222 billion. It is a stable, resilient, and robust sector, leading to employment in the EU for 4.5 million people. It is worth mentioning that the contribution of the food and drink industry to the gross domestic product of the EU stood at 1.9% in 2018 [2].

Within the agri-food industry, the ones associated with the transformation of vegetables (processing of vegetable raw material by means of any preservation technique) are grouped. In 2019, processed fruit and vegetables were worth EUR 51.5 billion, representing 6.5% of the overall value of the EU food industry’s output [3].

It is estimated that about 22% of the total food losses and wastes produced by the food chain worldwide are fruits and vegetables [4]. It should be noted that for every ton of fresh product, about 10–60% correspond to processing losses [5], representing approximately six million tons of solid waste and by-products every year in the EU [6].

As a consequence of the sorting, peeling, cutting, sizing, and packaging operations required for the production of canned, frozen, and ready-to-serve meals, these wastes, known as by-products, are generated. These are vegetable raw materials such as stems, leaves, skins, and products discarded because of size, color, shape, etc. These are rich in carbohydrates, proteins, minerals, and fiber [5] and excellent sources of vitamins and minerals, antioxidants, and diverse bioactive compounds [7]. Therefore, their potential for other applications is very relevant. One of the most interesting alternatives that deserves greater attention is their inclusion as animal feedstuff [8,9].

Vegetable by-products are characterized by a high moisture content, which often exceeds 80%, hindering their handling and accelerating microbiological growth [10]. In addition, their availability is often limited by their seasonal production [11]. Furthermore, other concepts that must be studied to evaluate their potential as animal feedstuffs are their nutritional value [12,13] and digestibility [12,14,15], which are key factors when including them in a ration.

Ruminants, due to their rumen microbiota, have the unique capacity to utilize fiber [16]. Thus, the introduction of vegetable by-products into the ruminant diet can convert them into high-quality products available to humans, such as milk or meat, promoting sustainable agricultural systems [17]. Subsequently, cereals might be largely replaced by these by-products [16]. As an example, intensive beef production systems in the EU are based on high-concentrate diets, maintaining low forage/concentrate ratios. More specifically, in Mediterranean systems, the area of the present study, cattle are usually fed with a resulting concentrate-to-straw ratio of 90:10 [18]. Therefore, if the EU has produced, in the last 5 years (2018–2022), 7.72 million heads of young calves [19] fed mostly on a conventional diet, the possibility of improvement is considerable.

Moreover, by introducing vegetable by-product-based diets in animal feeding, the competition between human and animal nutrition can be decreased. Taking into account the growing world population (projected as 10 billion by 2050 [20]), with 820 billion people suffering hunger in 2018 and over 2 billion people suffering moderate to severe food insecurity in the world [11], it is a must to make the best possible use of the resources obtained from the field by applying circular economy principles.

In order to close the loop, introducing by-products to different production lines adds great value when these are produced locally [21], promoting rural development in the areas where they are generated and achieving economic, social, and environmental benefits [21,22]. In addition, animal feed is the largest single cost item of livestock production, accounting for 60–70% of the total cost [23], and as the current global situation associated with high prices and stock/supply problems makes operations unpredictable, the usage of vegetable by-products can reduce external dependence, improving the economic profitability of livestock.

For all the above-mentioned reasons, the aim of the present study was, first, to evaluate the nutritional value of different vegetable by-products, by means of chemical composition*,* in vitro digestibility, and fermentation kinetics, and subsequently, to further evaluate their application in the formulation of a sustainable fattening calf ration.

## 2. Materials and Methods

**Figure 1:**
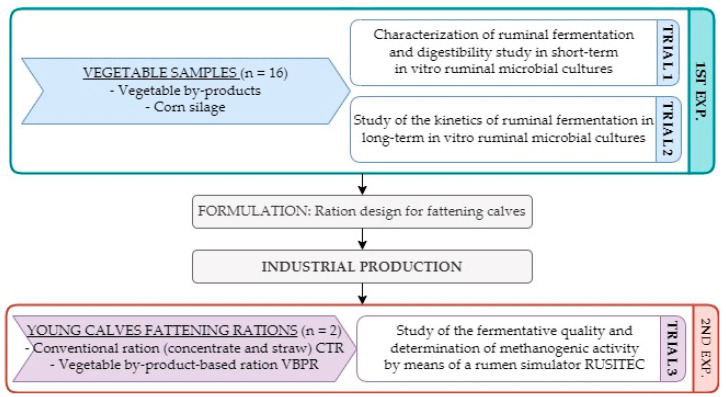
Visual scheme of the experiments and trials performed.

The study comprised two main experiments (Figure 1). The first experiment consisted of two different batch fermentation trials. The first one was a short-term in vitro batch fermentation trial designed to determine in vitro organic matter digestibility (IVOMD) of vegetable by-products. Corn silage was used as a reference raw material (positive control) (Trial 1). With the same samples, a long-term in vitro batch fermentation trial was also carried out to study differences in fermentation kinetics (Trial 2). The second experiment consisted of comparing a complete ration intended for calf fattening using vegetable by-products against a conventional ration composed of concentrate feed and straw, using an artificial rumen (Rusitec) (Trial 3).

### 2.1. Vegetable By-Products

Vegetable by-products (generated by the frozen industry) and corn silage were obtained from a by-products management company (Tratamiento Subproductos Agroalimentarios, S.L.; TRASA, Navarra, Spain). A total of 16 different samples were used in the experiments: 15 vegetable by-products and 1 corn silage. From the vegetable by-products, there were analysed a total of 10 main unitary vegetables that are generated in high volume (beans, broccoli, carrot, cauliflower, chickpea, green bean, peas, pepper, potato, and spinach), and 5 by-product mixes were selected mimicking the real examples of by-product generation of the frozen agri-food industry in Navarra. The composition of the 5 mixes was established by individual weighing of different vegetables on a fresh matter basis. The final weight of each mix was 500 g and individual weights are reported in Table 1. Samples were cut, dried (with a forced-air oven at 60 °C for 48 h), and finely ground (to pass a 1-mm screen). Chemical characterisation of the 16 samples was also carried out.

### 2.2. Short-Term In Vitro Batch Fermentation Trial (Trial 1)

The 16 samples served as substrates in four in vitro runs that took place in four different weeks. In each incubation run, rumen content was collected from a multiparous Latxa ewe fed *ad libitum* a basal diet (80% meadow hay and 20% concentrate) for 3 weeks and had free access to fresh water and feed. Ruminal content was collected after slaughter (before offering the morning feeding) and filtered through four layers of cheesecloth into a volumetric flask. Then, the rumen fluid was diluted in culture medium in a 1:4 ratio (ruminal fluid and phosphate-bicarbonate buffer [24], respectively) under anaerobic conditions.

In each run, approximately 500 mg of each sample were weighed into 125 mL serum bottles in triplicate. Then, 50 mL of culture medium was added. The bottles were crimp-sealed and incubated at a constant temperature (39 °C) in an incubator for 24 h. Gas production was released at 2, 4, 6, and 22 h post-inoculation to avoid pressure in the bottle headspace exceeding 48 kPa, as suggested by Theodorou et al. [25]. After 24 h of incubation, bottles were put at −20 °C for 20 min to stop fermentation for subsequent sampling of short-chain fatty acid (SCFA) and IVOMD determination.

IVOMD was calculated as described by Pell and Schofield [26], where 45 mL of a neutral detergent solution was added to each bottle and warmed at 105 °C for 1 h; then, the bottles were cooled, filtered through glass filter crucibles (Porosity 2) and washed with distilled water, ethanol, and acetone. The remaining sample was dried at 100 °C overnight and then burned in a muffle furnace at 525 °C to obtain true IVOMD values.

### 2.3. Long-Term In Vitro Batch Fermentation Trial (Trial 2)

Animals, substrates, and incubation procedures were the same as those described in the previous section. Approximately 1 g of each sample and 100 mL of culture medium were incubated for 96 h at 39 °C in 307 mL volume capacity glass bottles. The kinetics of gas production were recorded using the ANKOM^RF^ gas production system (ANKOM Technology, Macedon, NY, USA). Accumulated gas was automatically released through a valve attached to the module. The recording interval was set at 10 min, and a threshold of 1 psi for automatic release of accumulated gases to avoid supersaturation of CO_2_ in the medium and a valve opening time of 0.5 s were set in the ANKOM^RF^ software.

### 2.4. Complete Ration Design for Fattening Young Calves

From the results obtained in trials 1 and 2, together with the chemical characterization, a complete ration based on vegetable by-products was formulated. This ration was designed to meet the requirements for an average animal with a daily gain of 1.6 kg.

The vegetable by-products ration (VBPR) consisted of 53% concentrate, 37.5% vegetable by-products, 5.5% beet pulp, and 4% straw, on a fresh matter (FM) basis. This total mixture was ensiled in micro-silage units on an industrial scale by the TRASA company. The anaerobic fermentation that occurs in silage was used as a preservation technique to allow longer storage time and higher stability of the wet ration. In addition, a conventional fattening ration (control, CTR) consisting of concentrate and straw (90:10 ratio; FM basis) was used for comparison purposes. Both the concentrate and the straw were commercial raw materials for calf fattening. The ingredients and chemical composition of the two experimental rations, on a dry matter basis (DM), are given in Table 2. Samples of the two experimental diets were previously dried in a forced-air oven (60 °C, 48 h) and ground through a 2 mm sieve.

### 2.5. Rusitec Fermentation Trial (Trial 3)

The study was conducted using the Rusitec incubation procedure described by Czerkawski and Breckenridge [27]. The complete Rusitec unit consisted of eight fermentation vessels with an effective volume of 700 mL each. The inoculum was obtained from two slaughtered multiparous Latxa ewes. Before slaughter, ewes were fed an *ad libitum* basal diet (80% meadow hay and 20% concentrate feed) and fresh water for 3 weeks. Ruminal contents were strained through a double layer of cheesecloth to separate the solid and liquid fractions, and kept under CO_2_ flushing.

To begin the experiment, each fermentation vessel was filled with 400 mL of strained ruminal fluid and 300 mL of McDougall [28] artificial saliva. Then, 80 g of squeezed ruminal contents were weighted into a nylon bag (Ankom, 10 cm × 20 cm, pore size 50 mm, Macedon, NY, USA), which was placed inside the vessel together with a bag containing a total of 15 g of experimental substrate. After 24 h, the solid digesta bag was replaced by a new feed bag (experimental substrate). Thereafter, the bag that had remained 2 days in each vessel was replaced again by a new bag of feed (experimental substrate), so that each bag of feed remained in the vessel for 48 h. Fermentation vessels received a continuous infusion of artificial saliva at a rate of 600 mL/day for each vessel. Liquid effluent was collected daily in flasks containing 20 mL of H_2_SO_4_ solution (1:5, acid/water) to maintain pH below 2 in order to preserve fermentation products. This was performed for each experimental diet (VBPR and CTR) simultaneously.

A total of 15 g of the vegetable by-product-based feed was placed in each vessel daily. In the case of the control diet, straw and concentrate mixture (Table 2) was added (1.5 g of straw and 13.5 g of concentrate on a DM basis). Four fermentation vessels received the VBPR diet while another four vessels received the CTR diet. The incubation trial consisted of a 7-day adaptation period to achieve steady-state conditions, followed by a 4-day collection period.

During collection, once every 48 h, bags were removed from vessels, washed twice with artificial saliva, frozen, washed with tap water, and dried for DM disappearance, organic matter (OM), neutral detergent fibre (NDF), acid detergent fibre (ADF), starch (STA), and crude protein (CP) determinations. Samples from the liquid effluent (overflow flask) were collected daily for SCFA analysis. The gas produced was collected every 24 h in special gas sampling bags to determine the total gas production with a gas-flow meter (model DC-1, Shinagawa, Tokyo Japan). Gas samples were also collected in evacuated vials for later methane (CH_4_) analysis.

### 2.6. Chemical Analyses

Experimental substrates and designed rations were dried in a forced-air oven (60 °C, 48 h) and ground to pass a 1-mm screen. DM was determined by overnight drying at 103 °C (method 925.10) and OM content by charring at 525 °C for 24 h (methods 923.03) (AOAC 1990 [29]). Crude fat was determined by the Soxhlet system using diethyl ether as a solvent and with previous acid hydrolysis (method 920.39; AOAC 1990) [29]. CP content was measured using the Kjeldahl method 979.09, by AOAC 1994 [30]. Determination of NDF was performed following the method of Van Soest et al. [31], using α-amylase without sodium sulfite, and was expressed as free of ash. ADF and ADL were determined according to UNE-EN ISO 13906:2009 [32] by acid digestion and a subsequent charring at 525 °C. Gross energy (GE) was determined as the higher heating value (HHV) using an isoperibol LECO calorimeter (model AC500, Madrid, Spain) according to UNE-EN ISO 18125:2018 [33]. STA was measured using a polarimetric method [34].

The analysis of the SCFA (acetic, propionic, butyric, isobutyric, valeric, and isovaleric acids) was performed by gas chromatography (GC) using a flame ionization detector. A volume of 4 mL of rumen liquor mixed with 1 mL of a solution of 20 g/L of metyl-valeric acid as an internal standard in 0.5 N HCl. The mixture was centrifuged (15,000× *g* for 15 min at 4 °C) to separate the liquid phase from the feed residuals. After, the liquid phase was microfiltered (premium syringe filter regenerated cellulose, 0.45 µm 4 mm, Agilent Technologies, Madrid, Spain), and 0.5 µL of the liquid phase was directly injected in the GC (Agilent 6890 N, Agilent, Spain) using a capillary column (30 m × 0.53 mm i.d.; 1 µm film thickness; HP-FFAP, Agilent, Spain). Hydrogen was used as a carrier gas at a flow rate of 40 mL/min, the injection volume was 20 µL, and injector and detector ports were set at 300 °C. In the detector, air flow was 400 mL/min and make up (nitrogen) 25 mL/min.

Individual SCFA were identified using a standard solution of 4.50 g/L of acetic acid, 5.76 g/L of propionic acid, 7.02 g/L of butyric acid, 7.02 g/L isobutyric acid, 8.28 g/L of valeric acid, and 8.28 g/L isovaleric acid in 0.1 N H_2_SO_4_ (A6283, P1386, B103500, I1754, 240370, 129542, respectively; Sigma-Aldrich, Madrid, Spain). Quantification expressed in mmol/L was done using an external calibration curve based on the standards described above. Data were expressed in mmol/100 mmol.

Methane concentration in gas samples was measured in the GC explained for SCFA analyses (Agilent 6890 N, Agilent, Madrid, Spain) equipped with a capillary column (HP-FFAP polyethylene glycol TPA, 30 m × 0.53 mm i.d.), calibrated with a 10% CH_4_ standard, with a flux of 2 mL/min at 250 °C.

### 2.7. Calculations and Statistical Analysis

Digestible energy (DE) was calculated as proposed by the INRA system [35], multiplying GE values of individual samples by their IVOMD coefficient obtained in trial 1.

Gas volume estimates were obtained by correcting gas pressure values obtained in trial 2 by the substrate quantity in OM incubated. The gas pressure measured was converted to moles of gas produced using the ‘ideal’ gas law, and then converted to volume of gas produced using Avogadro’s law.

Fermentation kinetics were described according to the monophasic model described by Groot et al. [36]:

(1) $$G_{t}=\overset{n}{\underset{i=1}{\sum }}A_{i}\times {\left(1+{\;B}_{i}{}^{C_{i}}/t^{C_{i}}\right)}^{-1}$$

where G (mL/g OM) is the volume of gas produced per gram of OM incubated at time t after incubation; A*_i_* (mL/g OM) is the potential gas production; B*_i_* (h) is the time after incubation at which half of the potential amount of gas has been formed; and C*_i_* is a constant determining the sharpness of the curve.

The parameters A, C and B for each bottle were calculated using a non-linear regression procedure, which minimizes actual distances of data points to fitted curves by Marquardt’s algorithm.

From B and C parameters, the incubation time t_RM_ required for microbial fermentation and gas production to reach a maximum RM value (maximal gas production rate) were calculated from the following equations:

(2) $$t_{\mathrm{RM}}=B\times {\left(C-1\right)}^{1/C}$$

(3) $$\mathrm{RM}=\left(C\times t_{\mathrm{RM}}\right)/(B^{C}+t_{\mathrm{RM}}{}^{C})$$

The DM disappearance in trial 3 was determined by the weight difference between the samples prior to incubation and the bags obtained during the collection period after washing and drying. The disappearances of DM, OM, CP, NDF, ADF, and STA (weight difference) were measured in the same way, by the difference in the chemical composition results of the samples.

For trial 1, the total number of observations was 4 runs of processing × 16 in vitro incubation samples × 3 laboratory replicates = 192; however, after averaging laboratory replicates (incubation bottles), the remaining 64 observations were subjected to an analysis of variance using the GLM procedure of SAS [37] with the substrate as the fixed effect. Treatment means were separated using a Dunnet adjustment for multiple comparisons, with the corn silage defined as the control. Significant effects were declared at *p* ≤ 0.05.

For trial 2, the total number of observations was 4 runs of processing × 16 in vitro incubation samples = 64 observations that were subjected to an analysis of variance using the same statistical model described for trial 1.

For trial 3, the total number of observations was 2 in vitro incubation samples × 4 replicates in different fermentation vessels × 4 days of collection period = 32; however, after averaging over the four sampling days, the remaining 8 observations were analyzed by performing analysis of variance using the GLM procedure of SAS [37] according to the following statistical model:

(4) Yij = µ + Ti + εij

in which Yij represents the value of each individual observation, µ the average, Ti the effect of the ith treatment, and εij the residual error.

Treatment results are reported as least squares means. Significant differences between treatment and control were declared at *p* ≤ 0.05 using the Tukey’s multiple comparison test.

## 3. Results

### 3.1. Chemical Composition Analysis

The chemical composition and GE of corn silage and vegetable by-products are shown in Table 3. As expected, chemical composition was variable among the different raw materials. The DM content of vegetable by-products varied from 72.3 (cauliflower) to 392 g/kg (chickpea) in comparison with the DM content of corn silage of 369 g/kg. The OM content varied from 891 (pepper) to 977 g/kg DM (chickpea), although only three samples had an OM content below 925 g/kg DM (green bean, spinach, and pepper). CP varied widely, ranging from 120 (potato) to 379 g/kg DM (beans) in the vegetable by-products in comparison with the CP content of the corn silage (76.7 g/kg DM). In the same way, NDF content varied widely, from 162 (potato) to 418 g/kg DM (pepper), ADF from 61.0 (potato) to 446 g/kg DM (pepper), and ADL from undetectable values (chickpea and pea) to 142 g/kg DM (pepper). STA varied widely from undetectable values (carrot and cauliflower) to 588 g/kg DM (potato), in the case of corn silage the STA content was 363 g/kg DM. EE content was also variable, with the lowest value of 8.99 (potato) to 85 g/kg DM (mix 3 and spinach). Finally, GE contents among by-products ranged from 17.5 (potato) to 20.9 MJ/kg DM (pepper), being 18.2 MJ/kg DM in the case of corn silage.

### 3.2. In Vitro Digestibility and Fermentation Parameters of Corn Silage and Vegetable By-Products

Table 4 shows in vitro digestibility, digestible energy, and fermentation parameters of corn silage and vegetable by-products. Compared to corn silage (724 g/kg OM), all vegetable by-products, except for beans (822 g/kg OM) and pepper (694 g/kg OM; *p* > 0.1), showed a greater IVOMD, ranging from 994 g/kg OM (chickpea, *p <* 0.001) to 878 g/kg OM (spinach; *p <* 0.01). In addition, all vegetable by-products showed higher digestible energy compared to corn silage (13.8 MJ/kg OM), ranging from 20 MJ/kg OM in the case of chickpea (*p <* 0.001) to 16.3 MJ/kg OM in the case of pepper (*p <* 0.05).

Carrot (*p <* 0.1), chickpea (*p <* 0.05), green bean (*p <* 0.05), mix 1 (*p <* 0.1), mix 2 (*p <* 0.01), mix 3 (*p <* 0.01), mix 4 (*p <* 0.01), and mix 5 (*p* < 0.05) showed higher SCFA production than the corn silage. However, no differences were observed in SCFA related to truly digestible substrates, except for spinach which showed a lower value compared to corn silage (*p* = 0.033).

By studying the individual proportions of SCFA, no differences in acetic acid proportion (*p* = 0.953) were observed when comparing vegetable by-products and corn silage, but higher proportions of propionic acid were observed in chickpea and potato (*p* < 0.001), mix 2 (*p* < 0.01), pea, mix 3, and mix 4 (*p* < 0.05). However, lower concentrations were obtained in butyric acid for pepper and potato (*p* < 0.05) and for broccoli, pea, spinach, and mix 3 (*p* < 0.1). Therefore, there were no differences in the acetic to propionic ratio, and lower proportions in the acetic plus butyric to propionic ratio were observed for chickpea, potato (*p* = 0.05), mix 2, and mix 3 (*p* < 0.1).

There were no differences in isobutyric and valeric acid proportions (*p* > 0.1) and in total branched-chain fatty acids (BCFA) (*p* > 0.1), but spinach (*p* < 0.05) and pepper (*p* < 0.1) showed higher values of isovaleric acid compared to corn silage. As a consequence, there were no differences in the acetic to propionic ratio (*p* > 0.1), and lower proportions in the acetic plus butyric to propionic ratio were observed for chickpea and potato (*p* = 0.05), and for mix 2 and mix 3 (*p* < 0.1) compared to corn silage.

### 3.3. In Vitro Gas Production and Fermentation Kinetics of Corn Silage and Vegetable By-Products

Results from in vitro gas production kinetics are shown in Table 5, comparing vegetable by-products with corn silage according to the monophasic model described by Groot et al. [36]. For pepper, spinach, beans, and broccoli, lower potential gas production was obtained (*p* < 0.001), ranging from 119 mL/g OM (pepper) to 166 mL/g OM (broccoli) in comparison to corn silage (214 mL/g OM). However, in mix 4 (237 mL/g OM) and mix 2 (238 mL/g OM), a higher value of potential gas production was obtained (*p* < 0.1) compared to corn silage.

In the case of some vegetable by-products, less time was needed after incubation to reach half of the gas potential. Particularly, in a total of eight vegetable by-products (*p* < 0.001), this amount of gas was achieved between 8.21 (mix 3) and 10.2 h (mix5), compared to corn silage where 14.4 h were necessary. However, there were no significant differences in the shape of the gas production curve, except in the case of pepper (*p* < 0.05).

Less time to reach the maximum gas production rate value was needed in eleven vegetable by-product substrates, especially in the case of carrot and mix 3 (*p* < 0.001), with values of 8.01 and 8.89 h, compared to 15.8 h for corn silage. In addition, for broccoli, cauliflower, mix 1, mix 4, mix 5 (*p* < 0.01), and for green bean and mix 2 (*p* < 0.05). Finally, higher values were obtained for most vegetable by-products concerning the maximum gas production rate, with larger differences in the cases of broccoli, mix3, pepper, and chickpea (*p* < 0.001).

### 3.4. In Vitro Rumen Fermentation of Fattening Calves Rations in a Rusitec Trial

Nutrient disappearance values obtained in the Rusitec trial for the CTR and VBPR rations are shown in Table 6. The disappearance of DM, OM, ADF, and STA did not differ between VBPR and CTR rations. In contrast, VBPR had a higher disappearance of CP (*p* < 0.031) and lower disappearance of NDF (*p* < 0.057), compared with the CTR ration.

Rumen fermentation parameters with CTR and VBPR are reported in Table 7. Total SCFA production and the individual SCFA proportions did not differ significantly between the two experimental rations. In addition, non-significant differences between ration formulations were found for total gas production (*p* = 0.248), methane concentration (*p* = 0.594), or methane production (*p* = 0.712).

## 4. Discussion

Milk and meat consumption are expected to grow by 57% and 48%, respectively, between 2005 and 2050 [38]. This, linked with the fact that over one million people do not have a sufficient level of nutrition and a substantial change in land use [39], reducing the environmental impact of both the agri-food sector and livestock farming is crucial to improve the sustainability of them over the years [9]. It is worth mentioning that up to 40% of all arable land is used to produce animal feeds, resulting in a food-feed competition [40]. Thus, increasing food system circularity can help to reduce the pressure on the food chain. Specifically, if vegetable by-products generated in the agri-food industry are given a second life, and the 5% of by-products that are nowadays included in the global livestock feed ration [40] is increased, the environmental issue associated with their management will be minimized. Among the tools of circular economy is the destination of those by-products for ruminants, as novel feed resources able to reduce feed costs.

Prior to providing a ration containing a fraction of vegetable by-products, it is necessary to create technical knowledge about them. First, it is precise to determine the physicochemical composition and energy assessment. In the present work, the main vegetable by-products produced in the geographical location of the study were evaluated (The Ebro Valley, Spain). The results of the physicochemical composition are, in general, in agreement with previous studies. For instance, de Evan et al. [41] studied the potential of broccoli florets and stems as a novel feed. They obtained similar CP and EE concentrations, but lower values of ADF compared with those of the present study. For cauliflower sprouts, de Evan et al. [42] reported similar concentrations of CP, EE, and NDF. García-Rodriguez et al. [12] studied 26 agro-industrial by-products from Spain, including broccoli, green bean, pea, and pepper. Our results showed higher concentrations of CP and OM for broccoli, green bean, and pea, while pepper showed minor variations among the parameters. The differences in chemical composition compared to results reported in the literature might be due to the stage of growth, season, species and variety, soil types, and growth environment [43].

Moreover, with the aim of developing a real scenario, and going one step forward in the state of the art, mixes have been included in this study as they are real cases taking into account bagging operations in the vegetable freezing industry. The physicochemical characteristics of these mixes, as it was expected, reflected their composition in individual ingredients.

In addition to the physicochemical composition and energy assessment, it is necessary to know the fermentative characteristics of these by-products at the rumen level. With this objective, IVOMD and other parameters such as gas production and volatile fatty acid profile, as main fermentation products, were quantified and compared against corn silage, a common raw material in the livestock market. The major dissimilarity found was that the vegetable by-products were more degradable according to IVOMD, ED, and fermentation kinetics results. Values of IVOMD between 900 and 994 g/kg OM were obtained for 12 of the 15 vegetable by-product samples compared to 724 g/kg OM in corn silage. García-Rodriguez et al. [44] reported an even lower mean value of 670 g/kg for in vivo OM digestibility in 67 samples of corn silage. These results also indicate that most vegetable by-products were fermented more extensively and at a faster rate than the corn silage used in the present study.

Concentrations of ADL in vegetable by-products were relatively low, ranging between not detectable and 26 g/kg DM, except for pepper which was higher (142 g/kg DM), which agrees with the lower IVOMD and potential gas production of this by-product. Negative correlations of lignin concentration of forages with digestibility of forages have been reported for the last 50 years. Although this negative relationship is often reported for DM digestibility, lignin impacts cell wall digestibility and not digestion of the non-cell wall nutrients [45].

Based on their chemical composition, high IVOMD, DE, and fermentation kinetics, most vegetable by-products in this research can be considered as by-products with high-energy value, which can be incorporated in ruminant diets as replacements for other feedstuffs constituting the main energy source such as cereal grains. However, moisture concentrations of all by-products were high, ranging between 60 and 92%, and is also high for the vegetable mixes, ranging between 86 and 92%. The high water content has been known to negatively correlate with DM intake [46,47] and it is also related with microbial growth and feed spoilage. Therefore, it is necessary to stabilize these by-products using other methods such as ensilage, not only to reduce the moisture content but also to ensure their preservation and economic feasibility in order to facilitate their inclusion in animal diets.

Thanks to the use of the knowledge generated and going forward in the valorization of these vegetable by-products in animal feed, a real ration was formulated with the inclusion of vegetable by-products as ingredients in silages. In this sense, a ration for beef cattle fattening was proposed as an alternative diet to the conventional fattening system based on concentrates, which is mostly used in Europe. The aim was to reduce the consumption of concentrates and ingredients commonly imported from elsewhere by including a silage diet that includes locally generated vegetable by-products. To achieve this, the Rusitec trial was performed where the silage diet was compared to a conventional ration based on concentrate and straw (90:10) [18], with the objective of studying the behavior of both diets with the in vitro simulation of ruminal digestion conditions. The most significant differences in ingredient composition between the two diets were the inclusion of 8.2% of by-products, on a DM basis, and the reduction of the concentrate (12%) and straw (4.3%). However, very slight differences between diets were observed in the chemical composition and, therefore, those differences among both diets were too small to affect OM disappearance. The lack of effect in OM disappearance is in agreement with the absence of differences in SCFA (production and individual percentages), acetic to propionic ratio, and CH_4_ production. These results demonstrate that vegetable by-products ensiled with concentrate could be formulated for beef cattle finishing. Similarly, previous studies by Forwood et al. [48,49] reported that the incorporation of unsalable vegetables, concretely carrot and pumpkin, to be ensiled with crop sorghum has the potential to produce a sustainable, high-quality alternative ruminant feed.

In general terms, by including these vegetable by-products in the daily diet of livestock, the dependency on other ingredients can be considerably diminished, providing a more efficient and circular feeding system.

In order to advance in the generation of knowledge on this topic, further research is necessary, for instance, to quantify the environmental effect of the management of vegetable by-products to provide reliable information and facilitate policymaking in this area. Likewise, it would be necessary to extend knowledge to other rations for other livestock production systems.

## 5. Conclusions

Vegetable by-products from the agri-food industry used in this study can be considered as potential ingredients in ruminant rations. Most of the unitary vegetable by-products and all mixes, as real examples of by-product generation in the agri-food industry, have higher digestibility and a greater nutritional value than corn silage. An inclusion of 37.5% of ensiled vegetable by-products could be included in finishing cattle diets with no difference in ruminal fermentation. From here, it can be concluded that vegetable by-products could replace a considerable content of cereals and forages in beef cattle diets.

## Figures and Tables

**Table 1:** Individual composition of vegetable by-product in 5 mixes (g/kg of fresh matter) mimicking the real by-product generation of the frozen agri-food industry in Navarra.

	MIX1	MIX2	MIX3	MIX4	MIX5
Beans	53	0	93	0	71
Broccoli	227	0	126	179	244
Carrot	89	117	168	169	153
Cauliflower	0	0	0	227	209
Chickpea	54	0	0	0	43
Green bean	141	0	0	93	56
Peas	159	0	221	27	186
Pepper	0	215	0	0	0
Potato	192	431	229	305	0
Spinach	0	237	0	0	0
Others ^1^	85	0	163	0	38

^1^ Other minor components such as borage, rice, and sweet corn.

**Table 2:** Ingredients and chemical composition of the experimental rations.

Item	Treatment	
	CTR	VBPR
Ingredients composition (g/kg DM)		
Concentrate feed ^1^	896	776
Vegetable by-products ^2^	0	82
Beet pulp	0	81
Straw	104	61
Chemical composition (g/kg DM)		
DM (g/kg)	879	594
Organic matter	945	943
Crude protein	126	126
Neutral detergent fiber	259	231
Acid detergent fiber	114	116
Acid detergent lignin	14	19
Starch	448	424
Ether extract	69	64
Gross energy (MJ/kg DM) ^3^	19.1	18.8

CTR: control, VBPR: vegetable by-products ration, DM: dry matter. ^1^ Concentrate composition (DM basis); of 52.52% corn; 15% barley; 10% corn DDG; 8.42% soybean meal 47; 5% decorticated oat flakes; 3.06% palm oil; 3% soybean hulls; 1.6% calcium carbonate; 0.5% salt; 0.4% corrector; 0.35% sodium bicarbonate; 0.15% buffer in CTR ration; and 46.54% corn; 29.87% barley; 10.26% rapeseed meal 36; 7.69% corn dry distillers’ grains (DDG); 3.35% olive oil calcium soap; 1.14% calcium carbonate; 0.64% salt; 0.51% corrector in VBPR ration. ^2^ Vegetable by-products composition (DM basis) of 38.89% potato; 13.31% pea; 12.78% carrot; 10% broccoli; 8.7% beans; 5.23% spinach; 4.84% green bean; 2.46% chickpea; 1.63% cauliflower; 1.14% pepper; 1.03% others in VBPR ration. ^3^ Analyzed as higher heating value (HHV) by a isoperibol calorimeter LECO model AC500.

**Table 3:** Chemical composition (g/kg DM) and gross energy (MJ/kg DM) of corn silage and vegetable by-products.

	DM	OM	CP	NDF	ADF	ADL	STA	EE	GE ^1^
	(g/kg)	(g/kg DM)							(MJ/kg DM)
Corn silage	369	956	76.7	375	221	15.3	363	33.1	18.2
Beans	174	956	379	361	246	23.4	92.6	42.1	19.4
Broccoli	81.6	966	295	252	277	9.31	29.9	52.3	19.5
Carrot	94.4	933	129	200	193	4.61	nd	37.1	17.7
Cauliflower	72.3	947	264	214	217	4.34	nd	54.6	18.9
Chickpea	392	977	209	254	69.7	nd	466	81.3	19.7
Green bean	75.7	924	192	320	256	18.7	74.6	19.7	17.7
Pea	210	974	269	401	252	nd	110	44.3	19.1
Pepper	88.5	891	190	418	446	142	55.7	56.0	20.9
Potato	259	969	120	162	61.0	< 1	588	8.99	17.5
Spinach	102	895	342	265	155	25.8	66.8	84.8	18.4
Mix1	118	962	244	262	165	11.3	265	44.9	18.0
Mix2	140	946	140	180	103	10.5	461	23.5	17.6
Mix3	109	956	207	282	167	14.1	249	85.1	19.8
Mix4	106	953	135	184	108	3.58	381	20.4	17.7
Mix5	77.7	925	225	396	205	12.9	232	53.7	19.3

^1^ Analyzed as higher heating value (HHV) by isoperibol calorimeter LECO model AC500. DM: dry matter, OM: organic matter, CP: crude protein, NDF: neutral detergent fiber, ADF: acid detergent fiber, ADL: acid detergent lignin, STA: starch, EE: ether extract, GE: gross energy, Mix1 main components: broccoli, potato, pea, green bean, Mix2 main components: potato, spinach, pepper, carrot, Mix3 main components: potato, pea, carrot, broccoli, Mix4 main components: potato, cauliflower, broccoli, carrot, Mix5 main components: broccoli, cauliflower, pea, carrot, nd: not detectable.

**Table 4:** In vitro digestibility, digestible energy, and fermentation parameters of corn silage and vegetable by-products.

Substrates	IVOMD	DE	SCFA	SCFA: TDS	Individual SCFA Percentages (mmol/100 mmol)								
	(g/kg OM)	(MJ/kg OM)	(mmol/L)	(mmol/g OM)	Acetic	Propionic	Butyric	Isobutyric	Valeric	Isovaleric	BCFA	Ac/Pr	[Ac + Bu]/Pr
Corn silage	724	13.8	76.0	210	53.8	23.7	14.7	5.14	1.66	0.945	6.08	2.37	3.03
Beans	822	16.7 **	80.2	196	58.3	24.7	9.39	5.19	1.48	0.963	6.16	2.37	2.76
Broccoli	972 ***	19.6 ***	91.5	188	58.5	23.4	9.21 ^t^	5.98	1.55	1.38	7.36	2.55	2.95
Carrot	968 ***	18.4 ***	93.4 ^t^	193	54.8	28.7	10.1	3.78	1.98	0.701	4.48	1.94	2.29
Cauliflower	958 ***	19.1 ***	90.2	189	55.6	26.2	10.0	5.14	2.04	1.05	6.19	2.15	2.54
Chickpea	994 ***	20.0 ***	94.7 *	196	49.5	32.7 ***	10.9	3.93	1.94	0.921	4.86	1.55	1.88*
Green bean	922 ***	17.7 ***	94.0 *	204	56.9	25.3	10.5	4.59	1.83	0.952	5.54	2.30	2.72
Pea	906 ***	17.8 ***	88.2	195	52.1	30.2 *	8.62 ^t^	6.02	1.64	1.44	7.46	1.73	2.02
Pepper	694	16.3 *	72.6	209	57.9	25.0	7.76 *	6.37	1.52	1.52 ^t^	7.90	2.35	2.67
Potato	944 ***	17.1 **	92.0	194	50.5	34.9 ***	7.91 *	4.35	1.58	0.768	5.12	1.47	1.70 *
Spinach	878 **	18.1 ***	77.0	176 *	57.1	24.6	8.62 ^t^	6.44	1.72	1.55 *	7.99	2.35	2.70
Mix1	950 ***	17.8 ***	93.5 ^t^	197	54.2	29.1	9.37	4.61	1.71	0.965	5.58	1.89	2.22
Mix2	932 ***	17.3 ***	98.0 **	210	52.2	31.8 **	9.42	4.17	1.74	0.732	4.90	1.69	1.99 ^t^
Mix3	954 ***	19.8 ***	101 **	212	51.0	31.0 *	8.79 ^t^	5.76	2.18	1.32	7.09	1.67	1.96 ^t^
Mix4	967 ***	18.0 ***	101 **	208	51.0	30.9 *	11.7	3.74	2.02	0.711	4.45	1.69	2.07
Mix5	900 ***	18.8 ***	95.9 *	212	53.8	28.3	11.0	4.11	1.80	0.919	5.03	1.95	2.34
SEM	57.9	1.14	8.55	14.7	8.80	2.93	2.92	4.752	1.131	0.2849	4.604	0.504	0.540

IVOMD: in vitro organic matter digestibility, DE: digestible energy, SCFA: short chain fatty acid, TDS: truly digestible substrate, BCFA: branched-chain fatty acids, Ac/Pr: acetic/propionic, [Ac + Bu]/Pr: [acetic + butyric]/propionic, OM: organic matter, Mix1 main components: broccoli, potato, pea, green bean, Mix2 main components: potato, spinach, pepper, carrot, Mix3 main components: potato, pea, carrot, broccoli, Mix4 main components: potato, cauliflower, broccoli, carrot, Mix5 main components: broccoli, cauliflower, pea, carrot, SEM: standard error of the mean. Within a column, means differing from COS: ^t^: *p* < 0.1; *: *p* < 0.05; **: *p* < 0.01; ***: *p* < 0.001.

**Table 5:** In vitro gas production profile of corn silage and vegetable by-products.

Substrates	Gas Production Parameters ^1^				
	A (mL/g OM)	B (h)	C	tRM (h)	RM (h^−1^)
Corn silage	214	14.4	2.25	15.8	0.079
Beans	139 ***	12.8	2.42	14.6	0.096
Broccoli	166 ***	8.79 ***	2.63	10.4 **	0.154 ***
Carrot	208	9.02 ***	1.84	8.01 ***	0.104
Cauliflower	193	9.50 ***	2.19	10.2 **	0.116 *
Chickpea	230	11.1 **	2.74	13.5	0.132 ***
Green bean	200	9.95 ***	2.32	11.1 *	0.118 *
Pea	215	13.7	2.07	13.9	0.076
Pepper	119 ***	11.6 *	2.93*	14.4	0.133 ***
Potato	235	11.3 *	2.10	11.8 ^t^	0.095
Spinach	132 ***	11.0 **	2.22	11.8 ^t^	0.104
Mix1	209	9.87 ***	2.17	10.6 **	0.111 ^t^
Mix2	238 ^t^	10.8 **	2.08	10.9 *	0.099
Mix3	196	8.21 ***	2.22	8.89 ***	0.136 ***
Mix4	237 ^t^	9.61 ***	2.16	10.2 **	0.116 *
Mix5	215	10.2 ***	1.96	9.84 **	0.096
SEM	11.7	1.277	0.297	2.030	0.0164

A: the potential gas production, OM: organic matter, B: the time after incubation at which half of the potential amount of gas has been formed, C: constant that determines the sharpness of the curve, tRM: time to reach maximum gas production, RM: maximal gas production, Mix1 main components: broccoli, potato, pea, green bean, Mix2 main components: potato, spinach, pepper, carrot, Mix3 main components: potato, pea, carrot, broccoli, Mix4 main components: potato, cauliflower, broccoli, carrot, Mix5 main components: broccoli, cauliflower, pea, carrot, SEM: standard error of the mean. Within a column, means with the superscript differ from COS; ^t^: *p* < 0.1; *: *p* < 0.05; **: *p* < 0.01; ***: *p* < 0.001. ^1^ Monophasic model described by Groot et al. [36].

**Table 6:** In vitro ruminal nutrient disappearance of the experimental rations.

Item	Rations		SEM	*p*-Value
	CTR	VBPR		
Disappearance (g/kg DM)				
DM	744	764	28.5	0.359
Organic matter	753	760	29.8	0.762
Crude protein	612	695	42.0	0.031
Neutral detergent fiber	448	364	51.0	0.057
Acid detergent fiber	352	280	54.4	0.107
Starch	940	941	14.0	0.858

CTR: Control, VBPR: Vegetable By-Products Ration, SEM: standard error of the mean, DM: dry matter.

**Table 7:** Rumen fermentation parameters and gas production of the experimental rations.

Item	Rations		SEM	*p*-Value
	CTR	VBPR		
Fermentation parameters				
Total SCFA (mmol/day)	12.1	12.3	1.06	0.811
SCFA percentage (mmol/100 mmol)				
Acetic	50.4	49.2	3.94	0.677
Propionic	19.3	19.7	2.49	0.819
Butyric	19.7	21.1	1.24	0.169
Valeric	5.28	5.12	0.594	0.719
Isobutyric	0.903	0.921	0.0914	0.791
Isovaleric	4.40	3.96	0.471	0.236
BCFA	5.30	4.88	0.473	0.255
Acetic: propionic	2.63	2.68	0.356	0.866
Gas production				
Total gas production (mL/day)	2030	2233	471	0.248
Methane concentration (%)	4.08	3.92	0.826	0.594
Methane production (mL/day)	85.3	89.5	30.8	0.712

CTR: Control, VBPR: Vegetable By-Products Ration, SEM: standard error of the mean, SCFA: short chain fatty acid, BCFA: branched chain fatty acids, DM: dry matter.

## Data Availability

The data used in the study are available on request from the corresponding author.
